# Molecular Characterization Reveals Biodiversity and Biopotential of Rhizobacterial Isolates of *Bacillus* Spp

**DOI:** 10.1007/s00248-024-02397-w

**Published:** 2024-06-18

**Authors:** Alka Sagar, Shalini Rai, Sonia Sharma, Kahkashan Perveen, Najat A. Bukhari, R. Z. Sayyed, Andrea Mastinu

**Affiliations:** 1 Department of Microbiology and Biotechnology, Meerut Institute of Engineering and Technology, Meerut, India; 2 Department of Industrial Microbiology, Sam Higginbottom University of Agriculture, Technology and Sciences, Allahabad, 211007 India; 3 Department of Biotechnology, SHEPA, Varanasi, India; 4 Department of Botany & Microbiology, College of Science, King Saud University, P.O. Box-22452, 11495 Riyadh, Saudi Arabia; 5 Department of Microbiology, PSGVP Mandal’s S. I. Patil Arts, G B Patel Science and STKV Sangh Commerce College, Shahada, 425409 India; 6 Faculty of Health and Life Sciences, INTI International University, Persiaran Perdana BBN, Putra Nilai, 71800 Nilai, Negeri Sembilan Malaysia; 7 Department of Molecular and Translational Medicine, Division of Pharmacology, University of Brescia, 25123 Brescia, Italy

**Keywords:** *Bacillus*, Biocontrol, Phytohormone, Phytostimulant, Plant growth promotion, Siderophore

## Abstract

*Bacillus* species appearas the most attractive plant growth-promoting rhizobacteria (PGPR) and alternative to synthetic chemical pesticides. The present study examined the antagonistic potential of spore forming-*Bacilli* isolated from organic farm soil samples of Allahabad, India. Eighty-seven *Bacillus* strains were isolated and characterized based on their morphological, plant growth promoting traits and molecular characteristics. The diversity analysis used 16S-rDNA, BOX-element, and enterobacterial repetitive intergenic consensus. Two strains, PR30 and PR32, later identified as *Bacillus* sp., exhibited potent in vitro antagonistic activity against *Ralstonia solanaceorum*. These isolates produced copious amounts of multiple PGP traits, such as indole-3-acetic acid (40.0 and 54.5 μg/mL), phosphate solubilization index (PSI) (4.4 and 5.3), ammonia, siderophore (3 and 4 cm), and 1-aminocyclopropane-1-carboxylate deaminase (8.1and 9.2 μM/mg//h) and hydrogen cyanide. These isolates were subjected to the antibiotic sensitivity test. The two potent isolates based on the higher antagonistic and the best plant growth-promoting ability were selected for plant growth-promoting response studies in tomatoe, broccoli, and chickpea. In the pot study, *Bacillus subtilis* (PR30 and PR31) showed significant improvement in seed germination (27–34%), root length (20–50%), shoot length (20–40%), vigor index (50–75%), carotenoid content (0.543–1.733), and lycopene content (2.333–2.646 mg/100 g) in tomato, broccoli, and chickpea. The present study demonstrated the production of multiple plant growth-promoting traits by the isolates and their potential as effective bioinoculants for plant growth promotion and biocontrol of phytopathogens.

## Introduction

Bacteria of the genus *Bacillus* are soil-borne, endospore-forming, and stress-resistant bacteria from the phylum *Firmicutes*. They are ubiquitously present in many ecological conditions [1]. Gram-positive *Bacillus* species are the most promising plant growth-promoting rhizobacteria (PGPR), ecologically sound, and economically viable alternative to the pesticide usage in agriculture [2, 3]. These bacterial strains colonize the crop rhizosphere, efficiently suppress phytopathogens, and promote plant growth. Several researchers have reported the diversity, phylogeny, production, and secretion of degradative enzymes to combat phytopathogens [4], the production of a wide array of secondary metabolites and antibiotics [5, 6], and defense mechanisms like plant-induced systemic resistance [7, 8]. Some of the species of *Bacillus*aredescribed as endophytes that can promote plant growth using varied mechanisms, including colonization in roots, enlargement of root density, solubilization of minerals, enhanced nutrient uptake, and induced defense responses against abiotic and biotic factors [2, 9, 10]. Moreover, researchers employ *Bacillus* isolates that exhibit multifarious potentials such as phosphorus solubilization [11], production of indole-3-acetic acid (IAA) [12], siderophore [13], and 1-aminocyclopropane-1-carboxylate deaminase (ACCD) activity [14]. *Bacillus* species arewidely used biocontrol agents against various phytopathogens as commercially developed formulations are available [2, 15, 16].Commercially available forms of some *Bacillus* species include phytostimulants [17], biopesticides, and biofertilizers [18]. It has been widely used on various plants, including tobacco, soybean, cucumber, maize, rice, and watermelon [18–21].

Bacterial wilt caused by *Ralsatonia solanacearum* is a quarantine phytopathogen responsible for devastating agricultural losses worldwide [22]. It is a soil-borne phytopathogen that infects various commercial crops and can survive for long periods in the soil**.** During favorable conditions, the dormant bacterium is activated, enters through primary and secondary roots, and colonizes the host plants’ xylem vessels, leading to a lethal wilt disease. This disease causes enormous losses from the time of sowing till maturity. The disease is characterized by the appearance of yellow-colored leaves and light brown-colored lesions on the shoot androot, leading toreduced crop yield [22].

The present study evaluatedthe diversity, plant growth-promoting ability, and antagonistic activity of *Bacillus* sp. against *R. solanacearum* in tomato, broccoli, and chickpea.

## Materials and Methods

### Soil Sample

**Fig. 1:**
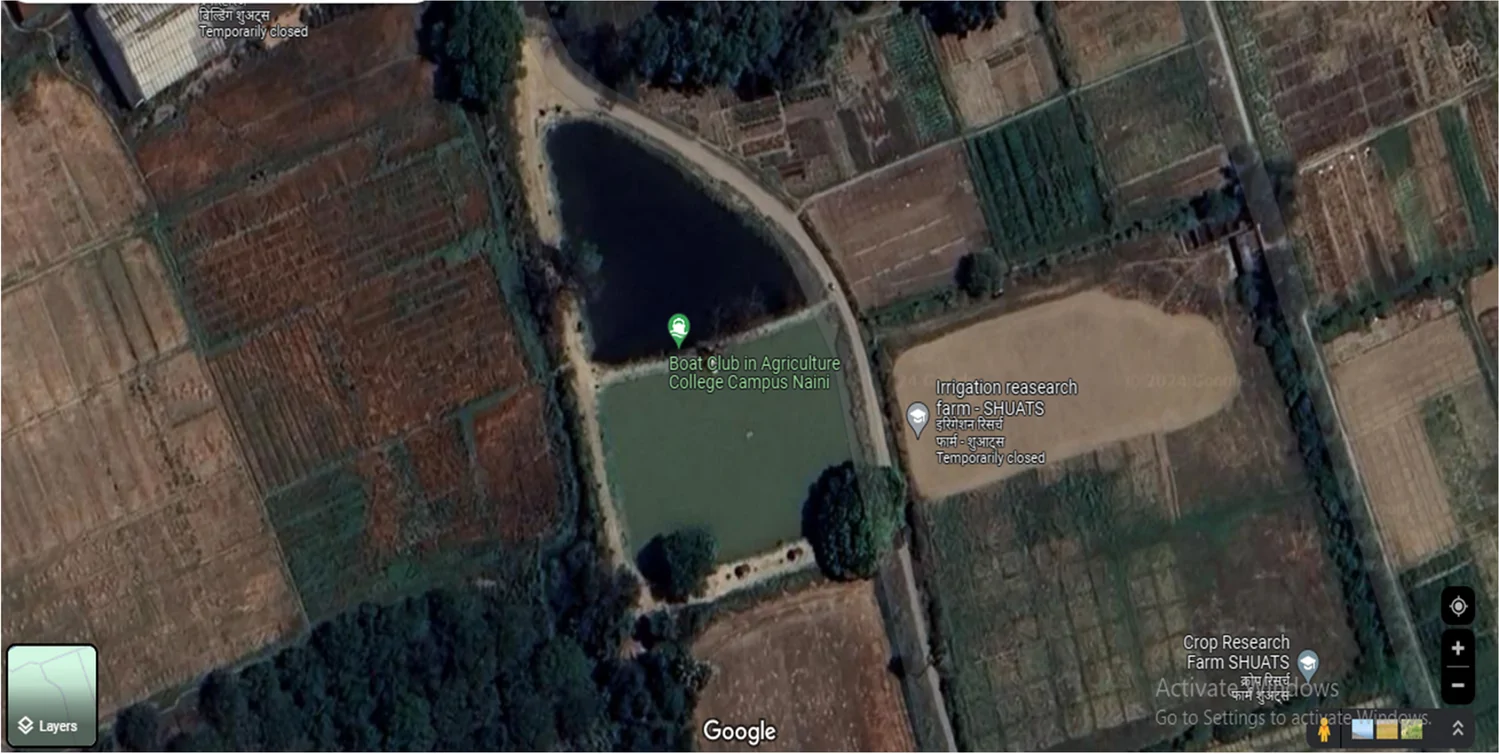
Map showing SHAUTS farm (Courtesy: Google map)

Ten grams of each sandy-loam soil sample were collected from the rhizosphere soil of tomato plants in Sam Higginbottom University of Agriculture, Technology and Sciences (SHUATS) Model Organic Farm (SMOF) Allahabad, India, (25° 24′ 42" N, 81° 50′ 56" E) [23, 24] (Fig. 1). Briefly, a rhizosphere soil sample (1 g) was transferred to 9 mL of sterilized phosphate buffer saline (PBS; 10 mL in 100 mL flask; pH 7.2) for 30 min, heated at 80 °C for 20 min in a water bath. The soil suspensions were serially diluted (10^–3^ to 10^–5^), and 0.1 mL of this suspension was spread on Nutrient Agar (NA) plates comprising methyl red (0.2%). The plates were incubated for 24 h at 30 °C. Single bacterial colonies were streaked onto fresh NA plates. Endospore staining was performed as described by Hamouda et al. [25].

### Screening of Antagonistic *Bacillus* Sp.

Seven selected *Bacillus* isolates were used for in vitro antagonistic activity using disc diffusion against *R. solanacearum* [26]. The growth inhibition of the pathogen was measured as the percent growth inhibition (PGI) using the following formula -

$$\text{PGI }=\frac{\mathrm{C}-\mathrm{T}}{\mathrm{C}} \times 100$$

where *C* = measure of control group growth and *T* = measure of treatment group.

The test was repeated 3 times with 5 independent replications.

### Molecular Identification of *Bacillus* Isolates

The standard microbiological protocols were followed for morphological and biochemical characterization of isolates. DNA extraction, partial 16S rRNA gene amplification, PCR product purification, and subsequent sequencing analysis of *Bacillus* isolates were performed as previously described [27]. The 16S rRNAgenewas amplified using universal primers, PF [5′-TGGCTCAGATTGAACGCTGGCGG-3′] and PR [5′-TGGCTCAGATTGAACGCTGGCGG-3′], and the PCR products were sequenced on ABI3100 Genetic Analyzer. The amplified sequences were run in the BLASTnprogram and compared with the NCBI database.

Using the multiple sequence alignment tool Clustal W, consensus sequences of the 16S rRNA gene from *Bacillus* isolates and reference sequences obtained from Genbank were aligned, using MEGA version 5 to contruct a phylogenetic analysis [28]. The unweighted pair group technique with the arithmetic mean (UPGMA) approach was used to conduct the analysis. The greatest composite likelihood approach was used to calculate the evolutionary distances, and the evolutionary history was constructed using the neighbor-joining method [29] with 2000 bootstrap replications, and the internal branches’ robustness was evaluated.

Molecular identification of *Bacillus* isolates was performed using rep-PCR with the help of BOXA1R ERIC-1R and ERIC-2F primers [28]. The PCR reaction mixture (25 μL) containing 5 × Gitschier buffer, 0.25 μM of primer, 50 ng DNA template, 1 U of Taq DNA polymerase (Bangalore Genie, India), and 2 mM MgCl_2_ was amplified on thermal Cycler (Biorad, CA, USA) and the amplified fragments were separated by agarose gel electrophoresis with the help of aDNA ladder of 100 bp to 3 kb.

### Screening for Plant Growth-Promoting Traits

#### Indole Acetic Acid Production

Indole acetic acid (IAA) production by the isolates was screened in Luria Bertani (LB) medium containing 1 g/L tryptophan [30].

#### Phosphate Solubilizing Activity

The inorganic phosphate solubilizing (PS) activities of bacterial isolates were perceived using the National Botanical Research Institute’s phosphate growth medium (NBRIP) agar medium at 37 ± 2 °C for 7 days. The abilityof the isolates to solubilize inorganic phosphate was calculated as a solubilization index (SI) using the following formula [31].

$$\mathrm{SI}=\frac{\text{Colony diameter }(\mathrm{mm}) +\text{ Zone diameter }(\mathrm{mm})}{\text{Colony diameter }(\mathrm{mm})}$$

#### Production of *Ammonia*

Isolates were screened for ammonia (NH_3_) production in peptone water (10 mL) inoculated and incubated for 48–72 h. Following the incubation, Nestler’s reagent (0.5 mL) was added, and the tubes were observed to color change from brown to yellow [32].

#### Siderophore Production

Screening of isolates for siderophore (SD) production was performed on Chrome Azurol agar (CAS) medium [33] at 30 °C. The isolates were grown on CAS agar medium at 30 °C for 48 h and observed for color change of medium from blue to orange/golden.

#### 1-Aminocyclopropane-1-Carboxylate Deaminase Activity

According to the available protocol, isolates were screened for 1-aminocyclopropane- 1-carboxylate deaminase (ACCD) activity [30]. The ACCD activity was defined as the amount of α-keto-butyrate produced per mg of protein per h.

#### Hydrogen Cyanide

Hydrogen cyanide (HCN) production by Bacillus isolates was estimated according to Lork’s protocol [34]**.**

#### Susceptibility to Antibiotics

*Bacillus* isolates were tested according to the method of Bauer [35] for its resistance to various antibiotics (Table 4). All assays were performed three times with five replications.

### Plant Growth Promotion Studies

#### In Vitro* Study*

The seeds of different vegetable crops such as tomato (variety-Lycopersicon; NTL-186), broccoli (variety-PalamSamridhi), and chickpea (variety- Pusa 256) were surface-sterilized with ethanol (70%) for 2 min and washed three times with sterile distilled water. The following treatments were applied.

T0- control (no bacterial inoculation),

T1 = *B. subtilis* PR30,

T2 = *B. subtilis* PR31.

The surface-sterilized seeds were transferred to bacterial suspension (10^4^ cfu/mL), kept for 60 min, placed on germination paper, and maintained for 15 days at 25 °C [24]. Control (T0) seeds without bacterial inoculation were used for comparison. Treated and control seeds were also checked for the root and shoot elongation pattern under in vitro conditions. All the treatments were performed in triplicates, and the average of triplicates was considered.

#### Percent Germination

The percent seed germination was calculated as follows [36].

$$\text{Seed germination }(\mathrm{\%})=\frac{\text{Number of germinated seeds}}{\text{Number of total seeds}}\times 100$$

#### Germination Index

The germination index (GI) was calculated according to the Association of Official Seed Analysts AOSA [37] using the following formula–

$$\mathrm{GI}=\frac{\mathrm{No}.\text{ of germinated seed }}{\text{Days of the first count}}+\frac{\mathrm{No}.\text{ of germinated seed}}{\text{Days of the final count}}$$

#### Vigor Index

Vigor index was calculated according to Abdul and Anderson [38] and with the help of the following formula**.**

$$\text{Vigor index} =\text{Percent germination }\times \text{ Seedling length}$$

### Green-House Pot Experiment

#### Plant Seeds and Treatment

The bioefficacy of *Bacillus* isolates (PR30 and PR31) was evaluated under a greenhouse pot experiment at Sam Higginbottom University of Agriculture, Technology and Sciences (SHUATS), Allahabad, India. Surface sterilized broccoli, tomato, and chickpea seeds were treated with endospore suspension of *Bacillus* isolates (~ 10^8^ cells mL^−1^). Treated seeds were sown in plastic pots containing autoclaved field soil and watered daily. Control seeds were not treated with bacterial culture. Five seed pot^−1^ and 10 pot treatment^−1^ were maintained. The treatments for the bioefficacy experiment were scheduled as (1) *B. subtilis* PR30, (2) *B. subtilis* PR31, (3) and Control. Plant growth parameters were measured 30, 60, and 90 days after sowing (DAS).

#### Measurement of Plant Growth Parameters

Ten seedlings were harvested, root length, shoot length, fresh weight, and dry weight (mg/plant) were measured.The plant height (cm) was measured from the ground level to the growing tip of the main shoot, and the average height was calculated in cm. One plant was randomly selected from each pot, and the number of branches of each plant was measured and averaged.Flowers from three plants were counted and averaged. All the fruits from three selected plants from each replication of all the treatments were counted. All the fresh fruits from three selected plants from each replication of all the treatments were weighed after picking. Each replication’s average fresh fruit weight per plant (g) was recorded and subjected to statistical analysis. The diameter of the curd of three plants was measured at the widest circumference in cm, and the average diameter per curd from each pot was calculated. The Head or bud of three selected plants was weighted on electrical balance in g, and the average was found to give the bud weight per plant. The fresh weights of the three selected plants were recorded in each pot, and the average fresh weight was calculated. This calculated value was assumed as the average weight of the rest of the remaining plant per pot. The plants used to measure dry weight were also subjected to dry weight analysis. The plants were dried for 5–6 h at a temperature of 50–60 °C. The dry weights of all randomly selected plants in each pot were added, and the average was calculated.

#### Biochemical Parameters

##### Chlorophyll, Carotenoid, and Lycopene Content

Chlorophyll *a, b*, and carotenoid content were determined according to Arnon's method [39]. The absorbance of the resulting solution was read at 663, 645, and 480 nm for chlorophyll *a, b,* and carotenoids. The extraction and estimation of lycopene content was performed according to the method of Butnariuand Giuchici [40]. The absorbance of supernatant containing lycopene was read in a spectrophotometer at 472 nm. The total lycopene content was measured as lycopene mg per 100 g of fruit tissues**.**

$$\text{Lycopene }(\mathrm{mg}/100\text{ g}) =\frac{E}{3.45}\times \frac{20}{w}$$

where *E* = extinction coefficient; *W* = weight (g).

### Statistical Analysis

All the experiments were performed in triplicates, and the average valueswere calculated. The standard errors were calculated for all mean values and subjected to ANOVA followed by DMRT. The BOX and ERIC-PCR product cluster analysis was based on the binary matrix, presence (1), and absence (0) of the band for each strain. Principle component analysis (PCA) was performed using the XLSTAT software.

## Results

### Isolation and In Vitro Assessment of *Bacillus* Isolates

**Fig. 2:**
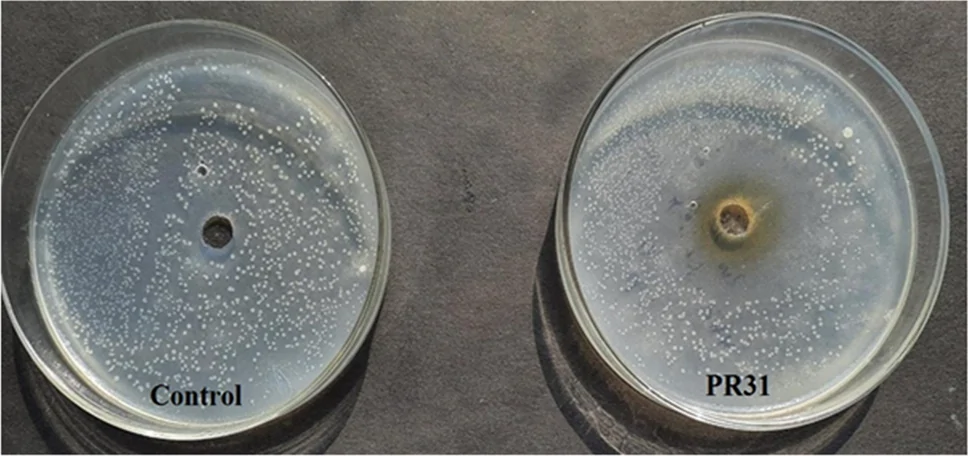
Antagonistic activity of isolated bacteria against *R. solanacearum*

A total 7 potent *Bacillus* isolates, (PR30, PR31, PR35, PR38, PR42, PR45, and PR55) were obtained from diverse soilsamples. These isolates showed antagonistic activity against *Ralstonia solanacearum* (Fig. 2) (Table 1).

**Table 1:** Antagonistic activity of isolated bacteriaagainst *Ralstonia solanaceorum*

Characteristics	PR30	PR31	PR35	PR38	PR42	PR45	PR48
Bacterial growth inhibition (%)	59.00^f^	63.29^ g^	30.51^a^	46.70^b^	49.04^bc^	54.63^e^	50.56^d^
Inhibition zone (mm)	16.90^e^	18.10^f^	6.70^a^	6.90^a^	8.66^b^	12.36^c^	13.96^d^

Values are the mean of three replicates. According to Duncan’s multiple range test, different letters in superscript are significantly different (*p* ≤ 0.05).

### Morphological and Biochemical Characterization of Strains

The *Bacillus*isolates were first characterized by their morphological and biochemical attributes. The isolateswere motile, spore-forming rods, forming white to creamy-white colonies, endospore former, tolerated pH (5–9), temperature (10–45 °C), and NaCl (0–10%) (Table 2).

**Table 2:** Morphological and biochemical characterization of antagonistic isolates of *Bacillus* sp. against *R.solanaceorum*

Characteristics	Isolates						
	PR30	PR31	PR35	PR38	PR42	PR45	PR48
Endospore	+	+	+	+	+	+	+
Colony colour	Off-white	Creamy white	White	White	White	White	White
Pigmentation	-	-	-	-	-	-	-
Motility	+	+	+	-	+	+	+
Salt (%)	0–10	0–10	0–8	0–8	0–9	0–8	0–8
pH	5–9	5–8	6–9	6–8	5–9	5–9	6–9
Temperature (°C)	15–45	15–45	15–55	15–45	10–45	15–45	15–45
Starch hydrolysis	+	+	+	+	+	+	+
Gelatin hydrolysis	+	+	-	-	-	+	+
H_2_S production	+	-	-	-	+	+	-
Glucose fermentation	+	+	+	+	-	+	+
Catalase	+	+	+	+	+	+	+
Oxidase	+	+	+	+	-	+	+
Indol	-	-	-	+	+	-	-
MR test	-	-	-	+	+	+	-
Citrate	+	+	+	-	-	-	+
Nitrate	+	-	-	-	-	+	-
Urease	+	+	-	-	-	+	+
VP test	+	+	+	-	-	-	+

-It was not determined.Values are the average of three replicates analyzed by Duncan’s multiple range test

They hydrolyzed starch and produced catalase. Three strains (PR30, PR42, and PR45) produced H_2_S, ammonia,siderophores IAA, HCN, and ACCD (Table 3). All strains showed intrinsic antibiotic resistance to variable levels (Table 4).

**Table 3:** Plant growth-promoting traits exhibited by the *Bacillus* isolates

Characteristics	PR30	PR31	PR35	PR38	PR42	PR45	PR48
Siderophore production	3.5 ± 0.05*	4.0 ± 0.04**	-	-	3 ± 0.09	3 ± 0.05	3.1 ± 0.06
Indole acetic acid	54.5 ± 0.05**	50.5 ± 0.03*	-	-	40.5 ± 0.15	41.5 ± 0.09	40.0 ± 0.15
Phosphorus solubilization activity	5.1 ± 0.05*	5.3 ± 0.02**	-	5.0 ± 0.03*	4.8 ± 0.06	4.4 ± 0.09	-
1-aminocyclopropane- 1-carboxylate deaminase	9.1 ± 0.05*	9.2 ± 0.07**	-	8.9 ± 0.76	8.5 ± 0.08	8.1 ± 0. 08	9.0 ± 0.16*
Ammonia production	+	+	-	-	+	+	+
Hydrogen cyanide production	+	+	-	-	-	-	+

+ , presence; -, absence. (% + SD) (low significant) *p < 0.05, (moderate significant) **p < 0.01, (high significant) ***p < 0.001. Values are the average of three replicates analyzed by Duncan’s multiplerange test

**Table 4:** Antibiotic resistance of antagonistic isolates of *Bacillus* sp

Characteristics	PR30	PR31	PR35	PR38	PR42	PR45	PR48
Ampicillin	+ B	+ C	-	+ B	+ B	+ C	+ B
Cephataxime	+ C	+ B	+ B	+ B	-	-	+ C
Nalidixic	+ B	+ B	-	-	+ B	+ B	+ C
Neomycin	+ B	-	-	+ B	+ C	+ B	+ C
Kanamycin	+ C	+ C	-	-	-	+ D	-
Tetracycline	+ C	+ C	-	+ B	-	+ B	-
Gentamycin	+ B	+ B	-	+ B	+ B	-	-
Chloramphenicol	+ C	+ C	+ B	+ B	-	+ D	-
Streptomycin	+ C	+ B	-	+ B	+ B	-	-

Nutrient agar media supplemented with antibiotics (µg/mL) represented by the different letters A = 20, B = 40, C = 60, D = 80, and E = 100. Values are the average of three replicates analyzed by Duncan’s multiple range test

### Identification of antagonistic *Bacillus* isolates

**Fig. 3:**
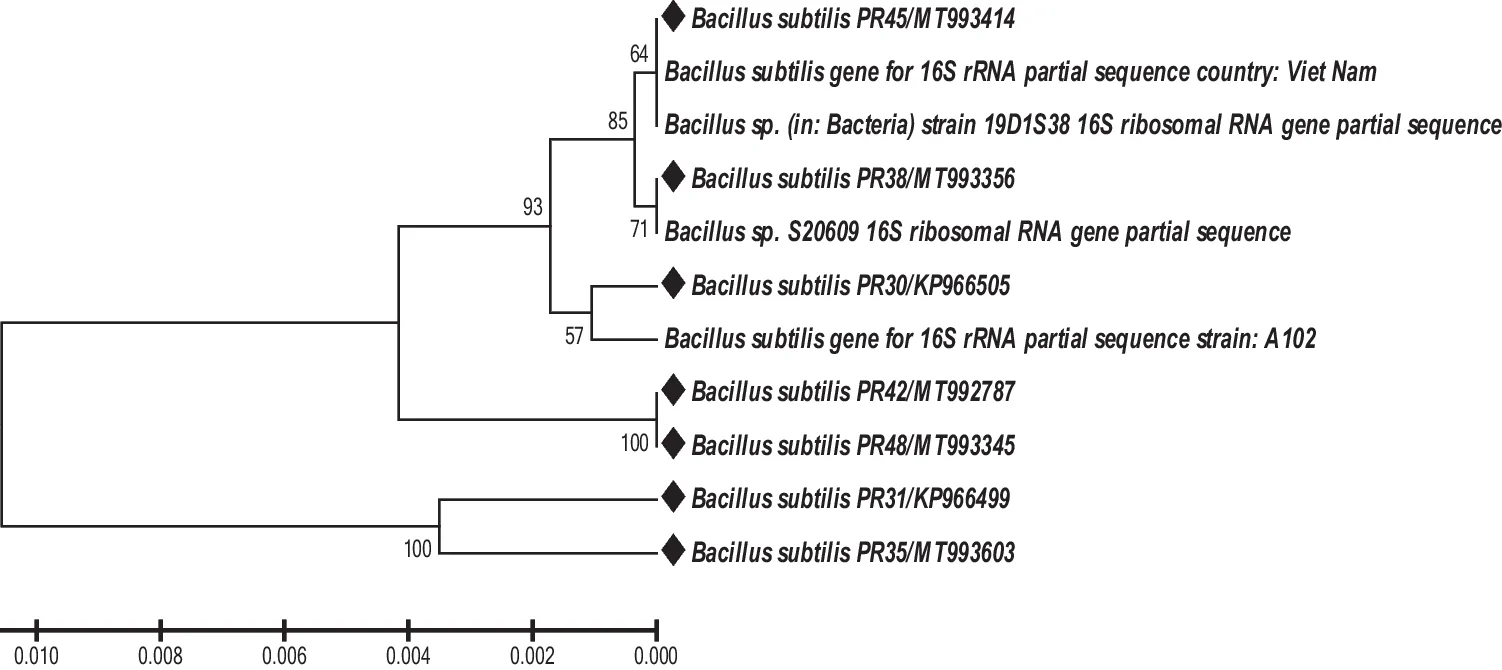
Phylogenetic tree based on the 16S rRNA gene sequence of potent *Bacillus* isolates were generated from organic farm soil samples based on the 16S rRNA gene sequence. Evolutionary distances were calculated using the “neighbor-joining” algorithm, based on a bootstrap analysis of 2000 replicates (values on branches denote % ofbootstrap support)

Seven *Bacillus*isolates were recognized as the active antagonists. Their phylogenetic distribution and 16S rRNA gene sequence individualities are presented in Table 5. The similarity values (≥ 97%) confirmed that all isolates (PR30, PR31, PR33, PR38, PR42, PR45, and PR48) belong to the genus *Bacillus subtilis*. The un-rooted phylogenetic tree (Fig. 3) presented the genotypic relationship of the isolates, wherever all the bacterial isolates were clustered into two major clades. The 16S-rRNA data showed that PR31and PR35 (*B. subtilis*) isolate was more diverse than other strains and grouped into distinct clusters.

**Table 5:** Identification of isolated bacteria based on 16S rRNA gene sequencing

Strain No.	Sample collection field	Sequence Size analyzed (bp)	Country	% Match with *Bacillus* sp.	NCBI accession No
PR30	Organic farm	1491	India	100	KP966505
PR31	Organic farm	1491	India	100	KP966499
PR35	Soil	1505	India	99	MT993603
PR38	Organic farm	1549	India	99	MT993356
PR42	Organic farm	1509	India	100	MT992787
PR45	Sugarcane field	1505	India	99	MT993414
PR48	Sugarcane field	1492	India	100	MT993345
A102	Plant	1503	China	98	AB526466.1
*Bacillus* sp. 19D1S38	Soil	1469	Korea	98	MN620404.1
*Bacillus* sp. S20609	Rhizosphere	1544	India	100	KF956597.1

### ERIC and BOX-PCR Analysis

**Fig. 4:**
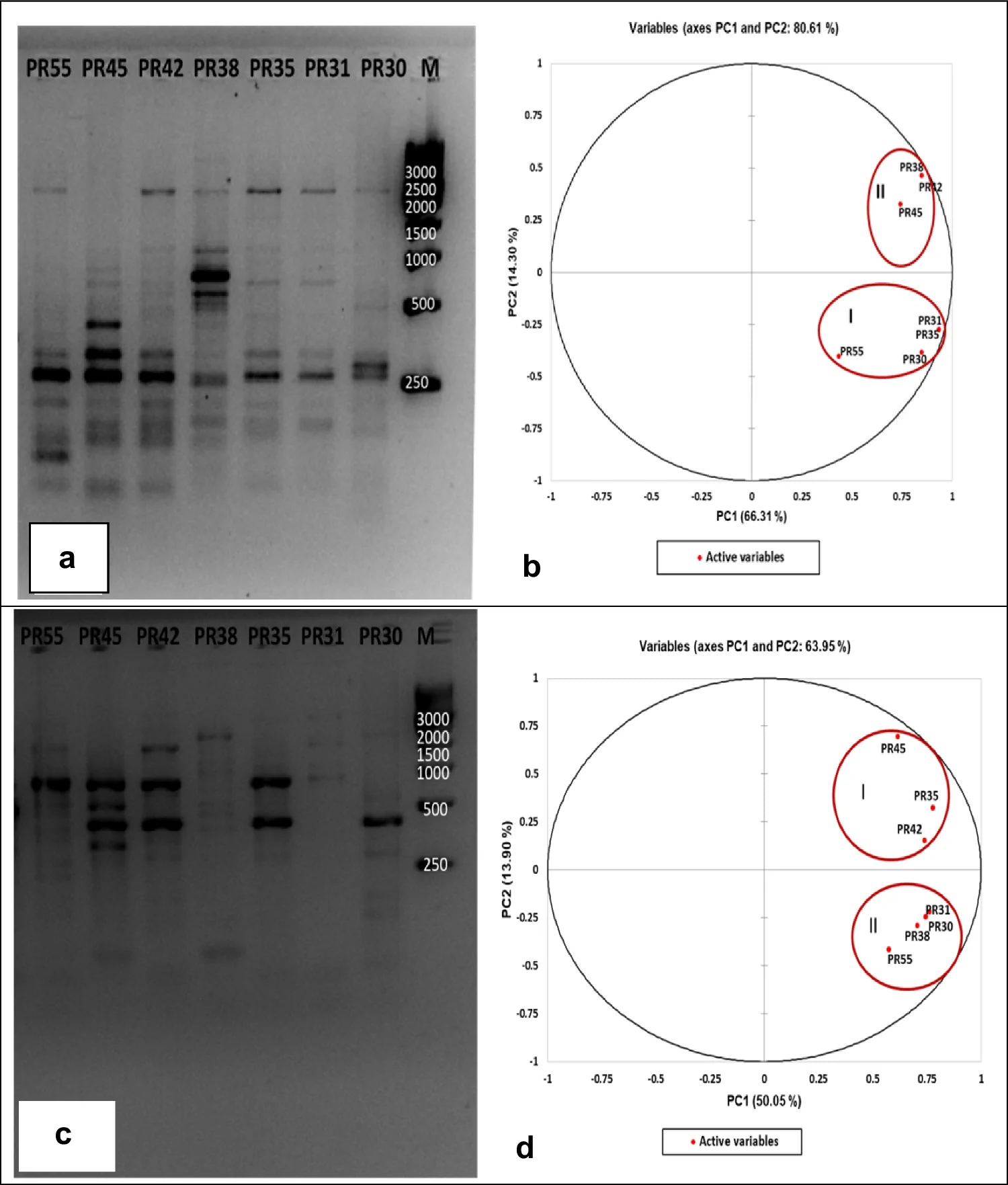
Genotypic patterns of bacterial strains obtained after ERIC (4a) and BOX-PCR (4b) fingerprinting. Principal component analysis score plot of seven bacterial isolates based on ERIC (4c) and BOX-PCR (4d) data

ERIC's complex fingerprint patterns produced 81 polymorphic bands of variable range (250–3000 bp) (Fig. 4a). Principal component analysis (PCA) based on the first and second coordinates showed a maximum Eigen value of 4.642 and minimum value of 0.095 with a percent variation of 66.31 and 14.30, respectively (Fig. 4b). Observation of the PCA analysis revealed that four isolates (PR30, PR31, PR35, and PR55) formed a major cluster (cluster I). Three isolates were classifiedin cluster II (PR38, PR42, and PR45).

The BOX-PCR banding pattern of all seven strains displayed 102 fragments in the 250–4000 bp (Fig. 4c). The results of the PCA analysis are based on the first and second coordinates and showed a maximum Eigen value of 3.053 and minimum value of 0.162 with a percentage variation of 50.05 and 13.90%, respectively (Fig. 4d). PCA analysis revealed that four isolates (PR30, PR31, PR38, and PR55) formed a major cluster (cluster II), and three isolates (PR35, PR42, and PR45) were classifiedin cluster I.

### Evaluation for Bioefficacy

Based on antagonistic activity and PGP traits, two isolates (PR30 and PR31) were selected for plant growth parameters of different vegetable crops under in vitro and greenhouse conditions. The *Bacillus* isolates (PR30 and PR31) induced a higher percentage (85–96%) of germination of seed compared to control seeds (Table 6).

**Table 6:** Inoculation effect of *B. subtillis* on percent seed germination and growth parameters of vegetable crops under in vitro condition

Treatments	Percent seed germination	Elongation in cm (± SD)		Index (± SD)	
		Root	Shoot	Germination	Vigor
Tomato (variety-NTLI86)					
**T0**	60 ± 1.1	4 ± 0.1	5 ± 0.1	1 ± 0.5	541 ± 1.5
**T1**	97 ± 1.5*	5 ± 0.3	7 ± 0.4	7 ± 0.5***	1141 ± 1*
**T2**	96 ± 1.7*	7 ± 0.4*	8 ± 0.4	9 ± 0.4***	1521 ± 1.0**
Broccoli (variety-PalamSamridhi)					
**T0**	70 ± 0.1	3 ± 0.1	4 ± 0.1	2 ± 0.1	490 ± 0.5
**T1**	94 ± 0.5*	5 ± 0.4	8 ± 0.4**	9 ± 0.5**	1172 ± 1.5**
**T2**	85 ± 0.5	7 ± 0.2*	9 ± 0.4**	7 ± 0.5**	1051 ± 1.0**
Chickpea(variety-Pusa-256)					
**T0**	60 ± 1.1	3 ± 0.1	4 ± 0.1	3 ± 0.4	421 ± 1
**T1**	93 ± 1*	5 ± 0.2	6 ± 0.4	7 ± 0.4*	992 ± 1.5*
**T2**	94 ± 0.5*	6 ± 0.3**	6 ± 0.4	8 ± 0.5*	1263 ± 1.0**

T0- control T1 = *B. subtilis* PR30, T2 = *B. subtilis* PR31 (% + SD) (low significant) *p < 0.05, (moderately significant), **p < 0.01, (highly significant),***p < 0.001. Values are the average of triplicates analyzed by Duncan’s multiple range test

**Fig. 5:**
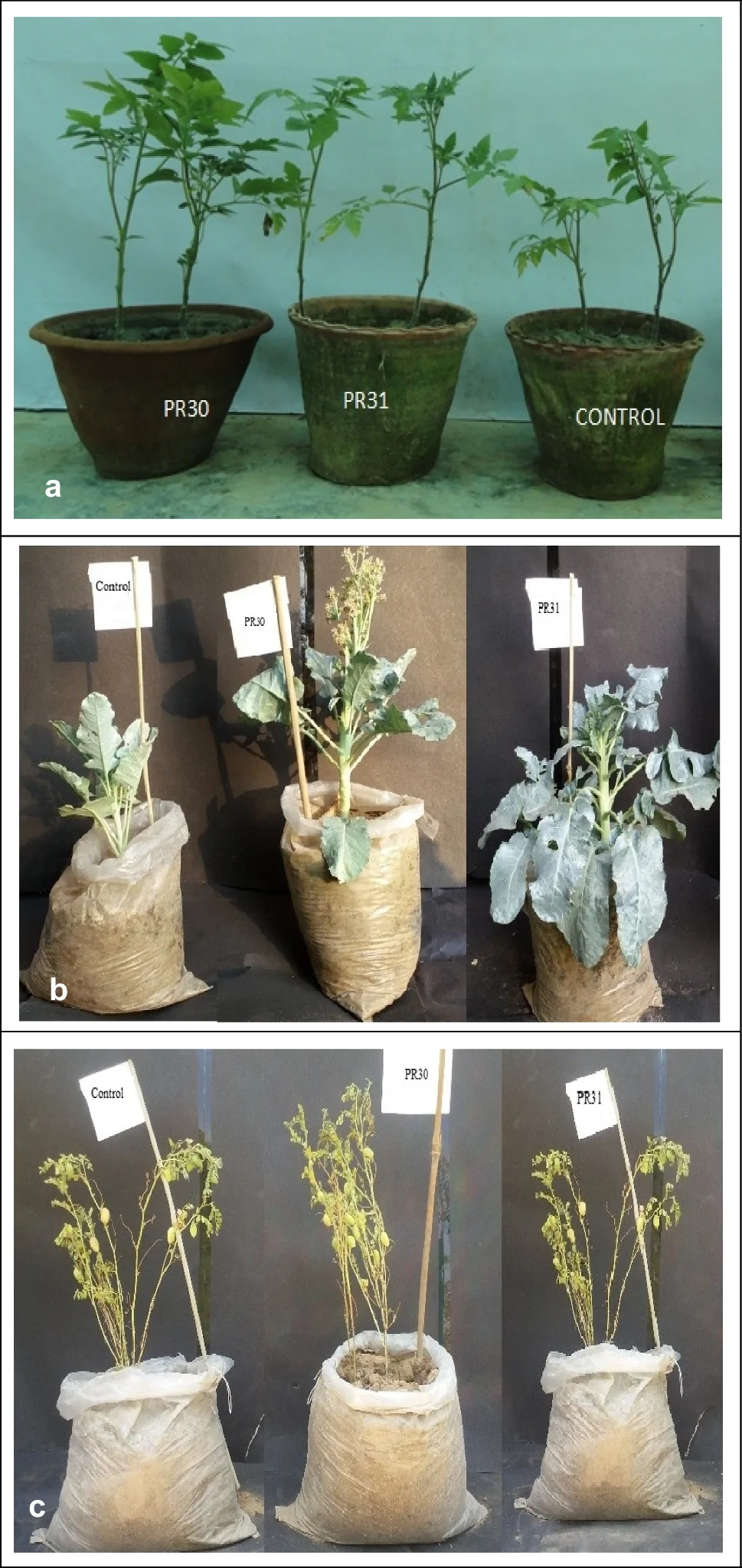
Plant growth promotion–greenhouse pot assay (5a). Tomato with control, (5b) Broccoli with control, and (5c). Chickpea with control

The *Bacillus* spp. isolates (PR30 and PR31) showed the best responses for plant growth promotion under greenhouse conditions. The efficacy of selected *Bacillus* isolates varied to induce germination, vigor index, and root and shoot length in tomato, broccoli, and chickpea (Table 6). Tomato seedling treatment with *B. subtilis* PR30 and *B. subtilis* PR31 significantly (*p* ≤ 0.05) enhanced seed germination (27%), root length (20–50%), shoot length (20–40%), fresh weight, and dry biomass (50–75%) compared to untreated (Fig. 5a) (Tables 6, 7). In the case of broccoli, a significant improvement in seed germination (15–24%), root length (40–60%), shoot length (50–60%), fresh weight, and dry biomass (50–60%) was evident over the control (Fig. 5b) (Tables 7 and 8). While this inoculation also improved seed germination (50–60%), root length (40–50%), shoot length (20–40%), and vigor index (50–80%) in chickpea (Fig. 5c). *Bacillus subtilis* PR30 and PR31 isolates significantly improved the chlorophyll and carotenoid content in tomato, chickpea, and broccoli, whereas lycopene was considerably enhanced in tomato plants (Table 8)**.**

**Table 7:** Effect of *Bacillus subtilis* on growth promotion of vegetable crops under greenhouse conditions

Treatments	Length (cm)		Fresh weight (g)		Dry weight (g)				
	Root	Shoot	Root	Shoot	Root	Shoot	No. of fruits/plant	Fresh fruit weight (g/plant)	Root colonization (log cfu/g root)
Tomato (variety-NTLI86)									
**T0**	15.47 ± 0.14	33.54 ± 0.17	6.901 ± 0.04	15.324 ± 0.10	2.042 ± 0.06	4.204 ± 0.06	7.30 ± 0.15	340.40 ± 9.18	-
**T1**	17.02 ± 0.09*	42.01 ± 0.32	7.891 ± 0.03**	19.022 ± 0.04	3.277 ± 0.13	4.950 ± 0.02	9.543 ± 0.11	393.16 ± 2.36	6.76 ± 0.08
**T2**	17.80 ± 0.07**	44.11 ± 0.15**	8.175 ± 0.03***	21.205 ± 0.29**	3.539 ± 0.02**	5.659 ± 0.08**	9.823 ± 0.10**	420.33 ± 0.60**	7.21 ± 0.13*
Broccoli (variety-PalamSamridhi)									
**T0**	8.51 ± 0.06	16.52 ± 0.05	31.666 ± 0.36	287.80 ± 1.07	5.410 ± 0.13	32.266 ± 0.17	4.22 ± 0.04	108.35 ± 0.17	-
**T1**	9.38 ± 0.09	18.62 ± 0.07*	38.270 ± 0.38	421.32 ± 0.98	6.690 ± 0.05	39.0567 ± 0.17*	5.76 ± 0.04	182.59 ± 0.40	6.17 ± 0.02
**T2**	9.99 ± 0.04**	19.41 ± 0.06**	41.026 ± 0.18**	4*45.45 ± 0.65*	7.680 ± 0.05**	40.670 ± 0.04**	6.61 ± 0.04**	196.28 ± 0.58**	6.72 ± 0.02*
Chickpea (variety-Pusa-256)									
**T0**	7.05 ± 0.32	16.51 ± 0.13	2.270 ± 0.04	8.210 ± 0.20	0.692 ± 0.04	1.651 ± 0.04	10.57 ± 0.04	25.37 ± 0.02	-
**T1**	10.51 ± 0.10**	22.86 ± 0.04**	2.947 ± 0.01***	10.076 ± 0.21**	0.984 ± 0.006**	2.133 ± 0.02**	12.99 ± 0.03**	31.86 ± 0.06***	6.92 ± 0.00*
**T2**	9.21 ± 0.16*	21.13 ± 0.15	2.530 ± 0.02	9.815 ± 0.02	0.743 ± 0.007	1.945 ± 0.02	12.42 ± 0.10	29.93 ± 0.07	6.57 ± 0.04

T0- control (no bacterial inoculation, T1 = *B. subtilis* PR30, T2 = *B. subtilis* PR31 Note: Mean ± SE value of three independent experiments. Each experiment was conducted in five replicates. *p < 0.05 = low significant, **p < 0.01 = moderately significant,,***p < 0.001 = highly significant. Values are the average of triplicates analyzed by Duncan’s multiple range test

**Table 8:** Effect of *Bacillus subtilis* on Biochemical traits of vegetable crops under greenhouse condition

Treatments	Biochemicals				
	Chlorophyll ‘a’(mg./g)FW	Chlorophyll ‘b’(mg./g) FW	Total chlorophyll (mg/g?	Carotenoid (mg/g) FW	Lycopene (mg/100 g)
Tomato (variety-NTLI86)					
T0	1.04 ± 0.004	0.733 ± 0.027	1.646 ± 0.950	0.806 ± 0.007	2.333 ± 0.027
T1	1.396 ± 0.051	1.083 ± 0.014	2.103 ± 1.214	1.243 ± 0.090	2.716 ± 0.082**
T2	1.413 ± 0.015**	1.236 ± 0.009**	2.493 ± 1.439*	1.313 ± 0.0381*	2.646 ± 0.060
Broccoli (variety-PalamSamridhi)					
T0	0.51 ± 0.020	1.696 ± 0.044	3.673 ± 0.030	0.533 ± 0.027	-
T1	0.666 ± 0.007	2.573 ± 0.094**	4.456 ± 0.126	0.686 ± 0.010	-
T2	0.686 ± 0.002*	2.376 ± 0.064	4.506 ± 0.072*	0.716 ± 0.015*	-
Chickpea(variety-Pusa-256)					
T0	1.09 ± 0.0478	0.36 ± 0.036	1.263 ± 0.051	0.543 ± 0.011	-
T1	1.643 ± 0.170	0.473 ± 0.0237**	1.6 ± 0.012	0.766 ± 0.0272	-
T2	1.766 ± 0.0272**	0.456 ± 0.0151	1.62 ± 0.024**	1.733 ± 0.0544**	-

T0- control (no bacterial inoculation, T1 = B. subtilis PR30, T2 = B. subtilis PR31. Values are the average of three replicates analyzed by Duncan’s multiple range test. Mean ± values are SD. (Low significant) *p < 0.05, (moderately significant), **p < 0.01, (highly significant),***p < 0.001. Values are the average of triplicates analyzed by Duncan’s multiple range test

## Discussion

Increasing agricultural productivity with limited cultivable land is the biggest challenge to growers around the globe. It is necessary to improve agricultural productivity to nourish and feed the growing world population. Crop yield and productivity can be enhanced in two ways: by increasing crop productivity through fertilizers or biofertilizers and by preventing crop losses caused by phytopathogens. Using PGPR that possesses the dual potential of plant growthpromotion and biocontrol is expected to play this dual role [24].

The diverse potential of *Bacillus* spp. makes it a promising plant growth-promoting rhizobacterium and BCA in various crops. Inoculation of crops with *Bacillus* spp. promotesseed germination, seedling vigor, leaf index, root and shoot growth, and photosynthetic ability. The plant growth promotion due to *Bacillus* spp. inoculation is due to the production of various PGP substances [2, 14]. Inhibition of phytopathogens results from the secretion of a wide range of antagonistic substances [17, 26]. Members of the *Bacillus* genus produce multiple PGP traits, such as phytohormones, and they help in nutrient mobilization (iron, P, etc.) [7, 14], which improves the growth of inoculated crop plants. *Bacillus* spp. is one of the major biological control agents (BCA) and antagonistic soil bacterium [21, 26]. *Bacillus* sp. produces various antagonistic substances, such as hydrogen cyanide, siderophore, and hydrolytic enzymes to inhibit the growth of phytopathogens.

Developing biofertilizers/formulation strategies using spore-forming *Bacillus*bioagents is an emerging area in crop protection. A total of 87 *Bacillus* strains were isolated from organic farm soil samples and examined by performing a disc diffusion approach to raise the possibility of using antagonistic bacteria asBCAs against *Ralstonia solanacearum*. Using this strategy, seven potent *B. subtilis* strains (PR30, PR31, PR35, PR38, PR42, PR45, and PR55) were specifically selected as an effective biocontrol agent against *R. solanacearum*. Similar strategies have been used effectively to isolate potential BCAs, such as *Bacillus* strains exhibiting antimicrobial activity towards phytopathogens [41].

Molecular identification based on 16S rRNA gene sequences of *Bacillus* isolates indicates phylogenetic clustering between bacteria at inter- and intra-species levels [42]. At the same time, the present study perceived that identification based on 16S rRNA gene sequences is limited and incapable of distinguishing along with bacterial strains. Consequently, polyphasic gene-based fingerprinting tools (BOX-PCR and ERIC-PCR] were used to discriminate intra-species unevenness between the bacterial strains. The results presented that isolates could not be distinguished by partial 16S rRNA gene sequence were different regarding ERIC and BOX-PCR patterns. Besides, these tools help separate the ecologically diverse *Bacillus* strains into a distinct group, which is otherwise tricky through 16S rRNA gene analysis [23].

Seedling treatment among isolates significantly suppressed the pathogen growth in in vitro conditions and enhanced plant height and biomass compared to control. Inhibition of phytopathogens is attributed to various antagonistic substances,viz*.*siderophore, hydrogen cyanide, other volatile compounds, and a wide range of antibiotics. Siderophore-producing PGPR prevents iron nutrition and, hence, the growth of phytopathogens [43]. HCN is a volatile organic compound (VOC) synthesized by a wide range of PGPR. Many bacterial genera, including *Bacillus*, can produce HCN [6, 20]. HCN exerts a potent toxic effect on many phytopathogens, forming stable complexes with Cu^2+^, Fe^2+^, and Mn^2+^ and causing disruption in protein functions [25, 44] (Reference?). It also inhibits electron transport and disrupts the energy supply to the cell, leading to living organisms’ death. Besides their biocontrol ability to produce HCN and antibiotics, *Bacillus* species produce phytohormones, increase uptake of phosphate and iron, produce ammonia, and protect cells from oxidative damage by producing catalase enzyme. Rahman et al. [43] reported the inhibition of *Agrobacterium tumefaciens*by HCN-producing *Bacillus megaterium* strain CtST3.5.

Many PGPR produce IAA, a crucial phytohormone associated with root elongation and initiation.Plants provide tryptophan to PGPR to synthesize IAA, an essential phytohormone for plant growth promotion. *Bacillus* spp. produced copious amounts of IAA, and this IAA has promoted root ramification.

Phosphorus is one of the vital elements for plant growth and development. It is regarded as a limiting nutrient for plant growth as it is usually present in insoluble forms. P solubilizing PGPR can potentially solubilize P and make it available forplant growth promotion [45].

Liu et al. [14] also suggested that the *B. amyloliquefaciens* strain displays maximum in vitro inhibitory activity towards multiple plant pathogens. Sudha et al. [45] reported the production of volatile compounds in *Streptomyces rochei* that inhibited the growth of sorghum pathogen. Sayyed and Patel [46] presented siderophore production in *Alcaligenesfaecalis* and found that this siderophoregenic culture inhibits the growth of a wide range of fungal phytopathogens. They found more antifungal activity in siderophoregenic culture than in a chemical fungicide. The present study also demonstrated that *B. subtilis* PR31 colonized more frequently than other test strains in tomato and broccoli rhizosphere, while *B. subtilis* PR30 was assessed more in chickpea. These findings justify the previous biocontrol reports that emphasize the proficient colonization of BCAs in the host rhizosphere,which is expected to enhance plant growth promotion and disease management.

Biocontrol efficacy of *Bacillus* spp. has been confirmed in the greenhouse and field conditions and at the post-harvest stage for fruit diseases [47]. It has been established primarily to resist gram-negative bacteria in vitro and under controlled conditions and to reduce diseases caused by these pathogens. A single strain can act against numerous bacterial pathogens. For example, *B. velezensis* LS69 has been shown to display antibacterial activities against *Erwinia carotovora* and *Ralstonia solanacearum* [43, 48]. Production of plant growth-promoting traits (PGPT) is the characteristic feature of all PGPR. These PGPT promote plant growth through direct mechanisms as green biostimulants [2, 49]. PGPR promotes plant growth through an indirect mechanism, such as the production of antibiotics [43] and the production of hydrolytic enzymes. The induction of resistance in plants and production of siderophore [46, 50] and phosphate solubilizing ability in different cultures of PGPR isolated from the rhizosphere have been reported**.** Kapadia et al. [51] reported the production of multiple plant growth-promoting traits in *Bacillus* sp., *Klebsiella variiocola*, and *Mesorhizobium* sp., respectively, and found that this multipotent culture improves growth in wheat and maize. The *Bacillus sutilis* isolates identified and used in the present study possessed all the plant growth promoting traits and hence are ideal candidates for biological control of *R. solanacearum.*

The production of ammonia is one of the major traits of PGPR that helps promote plant growth. The production of ACCD in PGPR is one of the best mechanisms involved in plant growth promotion under oxidative stress. PGPR lowers the ACC level in root exudates, decreasing the concentration of ethylene in the plant roots and thus helps in root length for better absorption of nutrients.Through antioxidants and other mechanisms, PGPR induces resistance to protect crop plants, thus helping plant growth [47, 48, 52–54].

*Bacillus* spp. suppressing the development of a wide range of phytopathogens while promoting plant growth in various crops can make this culture a multipotent PGPR for sustainable plant disease management and an eco-friendly biocontrol agent [2].

The present study successfully screened the *Bacillus* strains such as *B. subtilis* PR30 and *B. subtilis* PR31 associated with tomato rhizosphere that could stimulate growth in various crops (tomato, chickpea, and broccoli). These strains are identified as potent antagonists to suppress the growth of *R. solani*under in vitro conditions. They can be used in integrated disease management of tomato root rot and damping off. The combined studies, comprising biochemical and molecular technologies, are essential to select indigenous antagonistic *Bacillus* strains that can be used in combinations of other strains under different environmental conditions (greenhouse and field conditions to obtain resistance against pathogens in various crops). Without the extraordinary effect of genetic resistance in tomato cultivars, these biocontrols may be a potential candidate for handling vascular wilt infection and minimizing losses in enhanced fruit quality and yield. However, additional investigations are required to conclude the efficiency of these strains under diverse cultivar varieties and locations to understand the interaction behavior with the pathogen, host plants, and soil factors.

## Data Availability

No datasets were generated or analysed during the current study.

## Abbreviations

ACCD: 1-Aminocyclopropane-1-carboxylate deaminase
GI: Germination index
HCN: Hydrogen cyanide
IAA: Indole-3-acetic acid
MOF: Model Organic Farm
NA: Nutrient agar
PGI: Percent growth inhibition
PGP: Plant growth promoting
PGPR: Plant growth-promoting rhizobacteria
PSI: Phosphate solubilization index
SD: Siderophore
SHUATS: Higginbottom University of Agriculture, Technology and Sciences
